# Scorpion Venom and the Inflammatory Response

**DOI:** 10.1155/2010/903295

**Published:** 2010-03-14

**Authors:** Vera L. Petricevich

**Affiliations:** Laboratorio de Inflamación y Toxicología, Facultad de Medicina de la Universidad Autónoma del Estado de Morelos, Avenida Universidad 1001, Cuernavaca, Morelos 62209, Mexico

## Abstract

Scorpion venoms consist of a complex of several toxins that exhibit a wide range of biological properties and actions, as well as chemical compositions, toxicity, and pharmacokinetic and pharmacodynamic characteristics. These venoms are associated with high morbility and mortality, especially among children. Victims of envenoming by a scorpion suffer a variety of pathologies, involving mainly both sympathetic and parasympathetic stimulation as well as central manifestations such as irritability, hyperthermia, vomiting, profuse salivation, tremor, and convulsion. The clinical signs and symptoms observed in humans and experimental animals are related with an excessive systemic host inflammatory response to stings and stings, respectively. Although the pathophysiology of envenomation is complex and not yet fully understood, venom and immune responses are known to trigger the release of inflammatory mediators that are largely mediated by cytokines. In models of severe systemic inflammation produced by injection of high doses of venom or venoms products, the increase in production of proinflammatory cytokines significantly contributes to immunological imbalance, multiple organ dysfunction and death. The cytokines initiate a cascade of events that lead to illness behaviors such as fever, anorexia, and also physiological events in the host such as activation of vasodilatation, hypotension, and increased of vessel permeability.

## 1. Introduction

This review will focus on scorpion venom and its major toxins and their functions in excitable cells. It well also compares some of the research done on scorpions from different parts of the world to highlight open areas of interest.

## 2. Scorpions

Scorpions are venomous arthropods, members of *Arachnida* class and order *Scorpiones*. These animals are found in all continents except Antarctica, and are known to cause problems in tropical and subtropical regions. They are adapted to survive in a wide variety of habitats including tropical forests, rain forests, grasslands, savanna, temperate forests, caves, and even snow covered mountains. Actually these animals are represented by 16 families and approximately 1500 different species and subspecies which conserved their morphology almost unaltered [1, 2]. The scorpion species that present medically importance belonging to the family *Buthidae* are represented by the genera *Androctonus, Buthus, Mesobuthus, Buthotus, Parabuthus, *and *Leirus* located in North Africa, Asia, the Middle East, and India. *Centruroides spp *are located in Southwest of United States, Mexico, and Central America, while *Tityus spp *are found in Central and South America and Caribbean. In these different regions of the world the scorpionism is considered a public heath problem, with frequent statements that scorpion stings are dangerous. 

Accidents caused by scorpion stings are a relatively common event in subtropical and tropical countries and can cause lethal envenomation in humans, especially in children [3]. The signs of the scorpion envenomation are determined by the following: (a) scorpion species, (b) venom composition, and (c) the victim's physiological reaction to the venom. The symptoms of the sting start immediately with a few minutes after the sting and usually progress to a maximum severity within 5 hours. At this period the massive release of neurotransmitters results in sweating, nausea, and vomiting [4]. The victims usually have the major signs, with the most common being mydriasis, nystagmus, hypersalivation, dysphagia, and restlessness. They may exhibit signs and symptoms involving the central nervous system, stimulation of the autonomic nervous system, and occasionally, respiratory and heart failure, and even death. After stings by dangerous scorpions from different parts of the world the signs and symptoms are similar [5]. The victims of scorpion envenoming that presented multi-system-organ failure characterized by changes in hormonal environment with a massive release of counter-regulatory hormones, such as catecholamine, glucagon, cortisol, angiotensin-II, and with decreased levels of insulin and an increase blood glucose level. The grading of these scorpions envenomation depends on local signs and whether or not neurological signs predominant. 

The local signs observed in victims can present effects that can separate in a neurotoxic and cytotoxic local (Table 1). Central nervous system signs are sympathetic, parasympathetic, somatic, cranial, and peripheral nervous system and their major characteristics are shown in Table 2. The signs are also classified as nonneurological and neurological. The nonneurological signs which include cardiovascular, respiratory, gastrointestinal, genitourinary, hematological, and metabolic signs. With respect the neurological signs, most of the symptoms are due to either the release of catecholamine from the adrenal glands or the release of acetylcholine from postganglionic parasympathetic neurons [6]. Table 3 shows the summarized characteristics of the different grade of envenomation caused by scorpion venom.

## 3. Composition of Scorpion Venom

Scorpion uses its venom for both prey capture and defense. The venom is constituted by mucopolysaccharides, hyaluronidase, phospholipase, serotonin, histamine, enzyme inhibitors, and proteins namely neurotoxic peptides [7, 8]. 

The signs of the scorpion envenomation are determined by the symptoms presented by victims of scorpion envenomation are usually complex in nature and can be attributed mainly to hyperactivity of the autonomic nervous system [8]. The venom contain neurotoxic peptides which are responsible for the symptoms that present during envenomation by interacting with ion channels and have the potential to cause massive damage to nervous system of both vertebrates and invertebrates [4]. Ion channels are gated pores of which gating may be intrinsic or regulated by binding or changes in the voltage gradient. This gradient across is responsible by excitation of the nerve and muscle, hormonal secretion, cell proliferation, sensory transduction, the control of salt and water balance and regulation of blood pressure [9]. Scorpion toxins presents specificity and high affinity and have been used as pharmacological tools to characterize various receptor proteins involved in normal ion channel functionating, as abnormal channel functionating in disease-states [10, 11]. 

Advanced methods of fractionation, chromatography, and peptide sequencing have made it possible to characterize the components of scorpion, snakes and spiders venoms [12–14]. The venoms can be characterized by identification of peptide toxins analysis of the structure of the toxins and also have proven to be among the most and selective antagonists available for voltage-gated channels permeable to K^+^, Na^+^, and Ca^2+^ [15–17]. The neurotoxic peptides and small proteins lead to dysfunction and provoke pathophysiological actions, such as membrane destabilization, blocking of the central, and peripheral nervous systems or alteration of smooth or skeletal muscle activity [18]. 

The comparison of pharmacological characteristics with the different structures of scorpion toxins is important to understand and the mechanisms of action [17, 19, 20]. These toxins are responsible to overcome the defensive systems of the hosts such as proteases and/or significant local pH variations that may result from inflammation states induced by the animal bite itself [21, 22]. Scorpion toxins are classified according to their structure, mode of action, and binding site on different channels or channel subtypes [17–19]. Each class consists of several peptides isolated from the venom of different species of scorpion and are based on their pharmacological action and also agrees well with the structural properties of this peptide family. The long-chain toxins affecting sodium channels have been subdivided primarily into two major subtypes, *α*- and *β*-toxins [23, 24]. 

### 3.1. Scorpion *α*-Toxins

The *α*-toxins bind to receptor site 3 of the voltage-gated Na^+^ channels of vertebrates in a membrane-dependent manner [25]. Several studies have showed the effect and the biochemistry of these toxins. The major effects of *α*-toxins induce a prolongation of the action potential of nerves and muscles by fast inactivation of sodium channels receptor affinity dependent upon membrane potential [17, 19, 25–32].

### 3.2. Scorpion *β*-Toxins

The *β*-toxins are isolated from American scorpions, bind to receptor site 4 on vertebrate Na^+^ channels and producing a shift to a more negative membrane potential [33–38]. Several studies described the mode of action of these toxins that are related with the increment of sodium [36, 39–42]. The *β*-scorpion toxin CssIV obtained from *Centruroides suffussus* scorpion venom is believed to specific bind in sodium channel [23, 24, 33, 34]. Other toxin also described such as Ts1 is also known as Ts*γ* which is a major toxic component obtained of the venom from the Brazilian scorpion *Tityus serrulatus*. This toxin has also been classified as a *β*-scorpion toxin based on its structural homology, competitive binding assay, and its site of action [41–43]. The effect of these toxins has been described in different cell types [41–43].

The *β*-scorpion toxin is believed to bind, to only one of the four voltage sensors of the sodium channel [34, 36, 42, 44]. In accordance to the classical models of sodium channel gating, the voltage sensors of the sodium channel activate independently, and at least three of them have to be in an activated position for the channel to open [45–48]. However, if one of them is activated by the *β*-toxin, the threshold of activation is unlikely to shift significantly since other voltage sensors remain unaffected.

### 3.3. Sodium Channels Toxins (NaTx)

Voltage-gated sodium channels are critical for generation and propagation of action potentials initiation and propagation in excitable cells [25, 33, 49]. These channels are targeted for neurotoxins present a large variety of chemically distinct compounds that bind to several receptor sites on the pore-forming *α*-subunit [34, 35, 49]. With respect to scorpion toxins have been observed that they show a preference for distinct sodium channels subtypes of mammals or insects [20, 33–35, 50].

### 3.4. Potassium Channels Toxins (KTx)

Potassium channels are part of a large variety of biological processes and also are involved in an increasing number of human pathologies [9, 51]. The diversity of potassium channel blockers and their therapeutic value to overcome in the potential treatment of a number of specific human diseases specially autoimmune disorders, inflammatory neuropathies  and  cancer.  

Scorpion toxins that target K^+^channels (KTx) are short-chain peptides cross-linked by three or four disulfide bridges. The *α*-KTx family constitutes by more than 50 different *α*-KTx have been reported and listed in more than 18 families [18, 52–54]. Tenenholz et al. 2000 [55] described that *α*/*β* scaffold formed by an *α*-helix and a two- or three-stranted *β*-sheet linked by two bridges. However the *α*/*β* fold is shared by a variety of polypeptides with diverse functions, such as toxins active on Na^+^ channels [44]. The neurotoxin *α*-KTx 12.1 initially named as TsTX-IV was isolated from the *T. serrulatus* venom which is constituted with four disulfide-bridged described by several studies [18, 53, 56–59]. The voltage-gated potassium channel has been shown to play a role in immune responsiveness. Previous in vitro studies described that the blockade of the channel leads to diminution of T cell activation and delayed type hypersensitivity [60]. Butantoxin which is present in the venoms of three Brazilian scorpions *T. serrulatus, T. bahiensis*, and *T. stigmurus* has shown to reversibly block the potassium channels and inhibit the proliferation of T cells and IL-2 production [57].

### 3.5. Calcium Channel Toxins

Ca^2+^ ions play important roles in regulating a variety of cellular functions such as second messenger-coupling-receptor to active many cellular processes that including cellular excitability, neurotransmitter release, intracellular metabolism, and gene expression. The increment of Ca^2+^ concentration is mediated by voltage-gated Ca^2+^ channels that regulate Ca^2+^ influx across the plasma membrane and control the release of Ca^2+^ from intracellular stores. The Ca^2+^ channels are widely distributed in the body such as heart muscle, smooth muscle, skeletal muscle neurons, and endocrine cells [9, 25].

Scorpion venom consists of numerous peptides that may interfere with the activity of ion channels and modulate their functional properties. These peptides have different physiological and pharmacological activities. Various studies have been shown that scorpion toxins are used in insecticides, vaccines, cancer treatment, and protein engineering scaffolds.

## 4. Mediators Involved in Scorpion Envenomation

The inflammatory response is triggered by cascade that includes systems, cell elements, and release of mediators [61]. 

After exposure to antigen vertebrates respond by antibodies production through a series of events involving multiple cellular interaction. Initially, antigen presenting cells is recognized by a T cell antigen receptor. At the end, B cells produce antibodies that are able to specifically recognize the antigen that provoked their formation. In between the two antigen-specific events T cells help B cells to make antibody by producing cytokines and/or cell-cell interaction. The two subpopulations of Th cells which are named as Th1 and Th2 that differ in the effectors functions that differ mainly in the repertoire of cytokine secreted in response to antigenic stimulation [62, 63]. 

Th1 type cells secrete mainly interleukin-2 (IL-2), tumor necrosis factor (TNF-*α*), and interferon-*γ* (IFN-*γ*) which are responsible for macrophages activation and promote cell-mediated immune responses against invasive intracellular pathogens or the presence of toxins. Th1 cells also induce the production of IgG2a opsonizing antibodies [62]. The other subpopulation, Th2 type cells are important in the defense against extracellular parasites by induction of the IgE and IgA and produce a variety of cytokines such as IL-4, IL-5, IL-6, and IL-13 [62]. Both Th1 and Th2 cells secrete lesser amounts of TNF-*α*, granulocyte-macrophage colony-stimulating factor (GM-CSF), and IL-13. Mutual cross-inhibition between Th1 and Th2 type cytokines polarize functional Th cell responses into cell-mediated or humoral immune responses [62–67]. 

In accordance with their actions or properties the cytokines can be classified by pro-inflammatory or anti-inflammatory. Pro-inflammatory cytokines such as IL-1, IL-6, and TNF are primarily responsible for initiating an effective defense against exogenous pathogens. However, the overproduction of these mediators can be harmful and may ultimately lead to shock, multiple organ failure, and death (Table 4) [68, 69]. By contrary, anti-inflammatory cytokines include IL-4, IL-5, IL-6, and IL-10 are crucial for downregulating the exacerbated inflammatory process and maintaining homoeostasis for proper functionating of vital organs, but excessive anti-inflammatory response may also result in the suppression of body immune function (Table 4) [70–73]. 

The balance between pro- and anti-inflammatory activities determines the degree and extent of inflammation, and thus can lead to different clinical effects [74–76]. Anti-inflammatory cytokines counteract the effects of proinflammatory cytokines and therefore the relative concentration of a cytokine to its inhibitor or antagonist will determine its final effect. Cytokines imbalances mediate the development of organ damage and lethality during severe sepsis and envenomation, a lethal syndrome that can develop after infection or injury. Cytokine production by the immune system contributes importantly to both health and disease. Health requires that cytokine production is balanced; low levels are required to maintain homeostasis. Overproduction of some cytokines causes diseases that span the range of severity from mild to lethal. In addition, the setting of local infection or tissue injury, a well-adapted anti-inflammatory response may contain local inflammation and prevent it from evolving into systemic inflammation.

Systemic inflammation may be divided as acute or chronic; in acute systemic inflammation such as in sepsis, trauma, burns, and surgery is characterized by rapid and increments in plasma-levels of proinflammatory cytokines [77, 78]. In contrast, chronic low-grade inflammation is characterized by a modest but sustained increment in cytokines and acute phase reactants, usually of two to three folds and may be a key player in the pathogenesis of most chronic noncommunicable diseases [79, 80].

Much evidence supports the role of cytokines in scorpion envenomation seem that both pro- and anti-inflammatory cytokines levels are overproduced in sepsis syndrome. Their clinical significance and prognostic value have not been elucidated [76, 81–86].

The production of cytokines in the envenomation has previously been referred to as a cascade (Table 5). With respect to the pathogenesis of tissue injury is complex and cannot be attributed to a single agent. Tissue injury occurs during inflammation and is a progressive process which may eventually lead to organ dysfunction failure. The categorization of cytokines into pro- and anti-inflammatory response is essential for structural and functional repair of injured tissue, but excessive generation of proinflammatory signals can aggravate tissue damage because of the products derived from inflammatory cells.

### 4.1. Proinflammatory Cytokines

*IL-1* is a prototypic proinflammatory cytokine that exists in two forms named as IL-1*α* and IL-1*β*, both of which exert similar but not completely overlapping biological functions mediated through the IL-1 receptor (IL-1R). After binding to IL-1R, IL-1 induces the production of a high spectrum of cytokines and chemokines as well as the expression of adhesion molecules on endothelial cells, thus leading to the recruitment of inflammatory cells. In addition, IL-1 also contributes to the development of vascular damage by stimulating cell proliferation and differentiation and the release of matrix-degrading enzymes. IL-1R antagonist (IL-1Ra) is a structural homologue of IL-1 that binds to IL-1R but does not induce any cellular responses and is therefore a natural inhibitor of IL-1 activity [93]. Both are synthesized as precursor molecules (pro-IL-1*α* and pro-IL-1*β*) by many different cell types. Pro-IL-1*α* is biologically active and needs to be cleaved by calpain to generate the smaller mature protein. In contrast, pro-IL-1*β* is biologically inactive and requires enzymatic cleavage by caspase-1 in order to become active. IL-1*α* is primarily bound to the membrane, whereas IL-1*β* is secreted and thus represents the predominant extracellular form of IL-1 [94]. The levels of IL-1 in serum from human and mice injected with Brazilian scorpion *T. serrulatus* and/or its majors toxins are characterized by rapid increments of this proinflammatory cytokine. High levels of these cytokines were observed in supernatants of macrophage from mice exposed to *T. serrulatus* venom and its major toxins [91, 92]. Increased levels of IL-1*β* were determined in plasma from patients moderately or severely envenomed by *T. serrulatus* sting [82]. High levels of IL-1*α* and IL-1*β* were observed in sera from mice exposed to Mexican scorpion *Centruroides noxius* [76]. The role of IL-1 in scorpion envenomation has been investigated through influencing its level or activity. Table 5 shows the presence of this cytokine production after accident caused by different scorpions. 

*IL-6* is a multifunctional cytokine that regulates various aspects of the immune response, acute-phase reaction, and haematopoiesis. IL-6 is inducible by IL-1 and consequently concentrations in serum are often a reflection of IL-1 in vivo activity. Contrarily other cytokines, the IL-6 is a pleiotropic cytokine that exerts its proinflammatory effects is produced by a variety of cells including B and T lymphocytes, monocytes, fibroblasts, keratinocytes, endothelial cells, mesenchymal cells, and certain types of tumor cells. High levels of IL-6 were observed in sera from mice exposed to *Centruroides noxius *and *T. serrulatus *scorpion venoms [76, 84]. Increased levels of IL-6 were also observed in plasma from patients with different grade of envenomation by *T. serrulatus *[81, 82]. The cytokine production caused by different scorpions is described in Table 5. IL-6 is often used as marker for systemic activation of proinflammatory cytokines [95]. IL-6 has both pro- and anti-inflammatory effects. It downregulates the synthesis of IL-1 and TNF-*α* and also inhibits the production of GM-CSF, IFN-*γ*, and MIP-2 [95–98]. 

Scorpion venoms can stimulate the neuroendocrinal-immunological axis by its ability to release catecholamines, corticosteroids, bradykinin, and prostaglandins and all these agents proved to induce the release of immunological mediator cytokines. There is now accumulating evidence to suggest a causal relationship between overproduction of certain cytokines such as IL-1 and IL-6 and morbility and mortality associated with critically ill patients. Sofer 1995, [86], was the first that reported the involvement of the inflammatory systems after scorpion envenomation in humans. In this work is documented the increment of IL-6 levels in the serum of 8 of 10 children severely envenomed by the scorpions *L. quinquestriatus* and *B. judaicus*. The cytokines were measured on admission to the hospital and 1 to 3 hours after the sting IL-6 levels gradually returned to normal values at 12 and 24 hours measurements, but remained above control levels in all measurements. These results were quite similar to those found by others authors that described the cytokine production after sting caused by *Tityus serrulatus *scorpion in humans [81, 82]. With respect the experimental animal high levels of cytokines were found in serum from mice injected with *Centruroides noxius* and* T. serrulatus *venom [76, 84]. In all works the authors concluded that the activation and release of cytokines may play an important role in the pathophysiology of envenomation after stings and may be responsible for some systemic inflammatory manifestations and organ failure. More human and experimental animal studies are required to determine the contribution of the inflammatory system in the genesis of scorpion envenomation.

*IL-8* as pivotal mediator of cerebral reperfusion is increased in brain tissues and a neutralizing anti-IL-8 antibody significantly reduced brain oedema and infarct size in comparison to rabbits receiving a control antibody [99]. Increased levels of IL-8 were observed in serum from patients with different grade of envenomation caused by *T. serrulatus* and *Leiurus quinquestriatus *scorpions (Table 5) [82, 89, 90].

*TNF* is a pleiotropic cytokine that exerts potent proinflammatory effects on envenomed and other metabolic and inflammatory disorders which are also risk factors for cardiovascular diseases. TNF-*α* is primarily produced by monocytes and macrophages. Lymphocytes and macrophages orchestrate a lot of the inflammation in envenomation, mainly the production of TNF-*α* that exerts its proinflammatory effects through increased production of IL-1*β* and IL-6, expression of adhesion molecules, proliferation of fibroblasts, and procoagulant factors, as well as initiation of cytotoxic, apoptotic, and acute-phase responses [87, 100, 101]. TNF-*α* is a major inflammatory cytokine due to its ability to stimulate the synthesis of nitric oxide and other inflammatory mediators that drive chronically delayed hypersensitivity reaction [102, 103]. With respect to TNF-*α*, IL-1 seems to be important in the pathogenesis of envenoming because of its immunological upregulatory and proinflammatory activities [76, 84, 87]. The IL-1 system consists of IL-1*α* and IL-1*β*, both of which are produced by various cell types through the initiation of cyclooxygenase type 2, phospholipase A, and inducible nitric oxide synthase [102, 103]. High levels of TNF-*α* were observed in human and mice serum and also in mice macrophages supernatant (Table 5).

*IFN-* *γ*: the pleiotropic cytokine IFN-*γ* is a proinflammatory mediator that is expressed at high levels in envenomation by various cells, including monocytes/macrophages, Th 1 cells, and natural killer T cells (NK) [63]. High levels of IFN-*γ* were observed and documented during the envenomation caused in human and experimental animals by different scorpion venoms *Centruroides noxius *and *Tityus serrulatus* (Table 5) [76, 82–84].

### 4.2. Anti-Inflammatory Cytokines

*IL-4* is a highly pleiotropic cytokine that is able to influence Th cell differentiation, its early secretion leads to polarization of Th cell differentiation toward Th2 like cells [63]. Th2 cells secrete their own IL-4, and subsequent autocrine production of IL-4 supports cell proliferation. The Th2 cell secretion such as IL-4 and IL-10 has marked inhibitory effects on the expression and release of the inflammatory cytokines. IL-4 is considered to be able to block or suppress the monocyte-derived cytokines, including IL-1, IL-6, IL-8, TNF-*α*, and macrophage inflammatory protein 1 alpha [104, 105]. 

Overall anti-inflammatory cytokines whose roles are less well characterized in envenomation include IL-4 which has a stimulatory molecule for B and T cells, and has known immunosuppressive effects in the intestine [106]. T-cell receptor *α* chain-deficient mice (TCR-/-) treated with anti-IL-4 monoclonal antibody showed a decrease in Th2 cells mRNA cytokine production and an increase in expression of IFN-*γ*, suggesting that IL-4 plays a major role in inducing Th2 type CD4+ cells in the gut to shift towards a Th1 response [107]. Increment of IL-4 production was observed in serum from rats exposed to *Androctonus australis hector* scorpion [87] (Table 5).

*IL-10* is produced by several cell types including CD4+ and CD8+, T cells, macrophages, monocytes, B cells, dendritic cells, and epithelial cells [108]. IL-10 is the most important anti-inflammatory cytokine found within the human immune response. It is the potent inhibitor of Th1 cytokines, including IFN-*γ*, TNF-*α*, IL-1*β*, IL-2, IL-6, IL-8, and IL-12 [63, 108]. In addition, a major stimulus for the production of IL-10 is inflammation itself and IL-1*β* and TNF-*α* can stimulate IL-10 production directly [108]. The one other property of IL-10 is to suppress free oxygen radical release and nitric oxide activity of macrophages and the production of prostaglandins [109]. In serum from patients envenomated with *Tityus serrulatus* scorpions and in experimental animals exposed to *Androctonus australis hector, Centruroides noxius, *and *T. serrulatus *venoms were observed modified levels of IL-10 (Table 5).

The pathophysiology of envenomation is complex, but there is little doubt that injection often progresses from systemic inflammatory response to severe envenomation. In humans envenomed or experimental animal exposed to venom crude and/or purified toxins from different scorpions is the primary event in this sequence. During the interaction of venom components with cells and serum proteins to initiate a series of reactions that generally may lead to cell injury and death. The discovery that cytokines have the capacity to cause disease focused a new field of investigation on the physiological control mechanisms that maintain health by restraining on counter regulation cytokine release. Systemic effects of the cytokines have been shown to induce fever and increase symptoms. In the local action the cytokines promote recruitment of inflammatory cells to inflammation sites. In scorpion envenomation the balance between pro- and anti-inflammatory cytokines determines the degree and extent of inflammation, and thus can lead to different clinical effects. Anti-inflammatory cytokines counteract the effects of proinflammatory cytokines and therefore the relative concentration of a cytokine to its inhibitor or antagonist will determine its final effect. 

**Figure 1:**
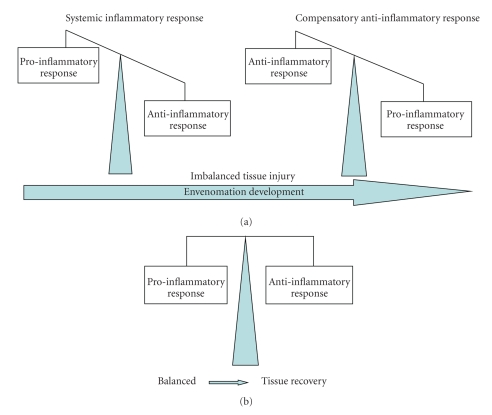
Categorization of cytokines pro- and anti-inflammatory based on their effects in envenoming models. (a) Imbalance between pro- and anti-inflammatory is crucial for envenomation development. (b) Balance of cytokines is crucial to tissue recovery.

Following the injection of venoms, a variety of proinflammatory cytokines are released, along with counter-regulatory or anti-inflammatory cytokines, and the outcome of an inflammatory response is dictated by a variety of factors, that including the duration of the stimulus, and the balance between the proinflammatory and anti-inflammatory response. An excessive proinflammatory response is thought to be important in the pathogenesis of septic shock. In contrast, an excessive anti-inflammatory response could result in failure to clear a venom action, with equally deleterious effects [73, 85, 110]. However the prolonged compensating anti-inflammatory response syndrome may be associated with excess mortality and morbidity because of increased risk for envenomation (Figure 1). With respect to the functions of many cytokines, and frequently multiple antagonists for any given agonist, the ability to compensate for a certain amount of divergence in production of individual cytokine is significant. Cytokines are important for regulation of inflammatory response. 

**Figure 2:**
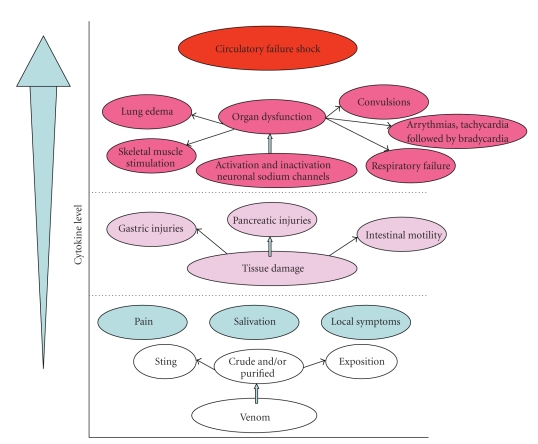
Effect of scorpion envenoming on cytokine production.

During the local and systemic responses are observed the release of proinflammatory cytokines, arachidonic acid metabolites proteins of the contact phase and coagulation system, complement factors; it is defined as systemic inflammatory response. The massive production of the cytokines causes that spam the range of severity mild to lethal. However, in parallel, anti-inflammatory mediators which produce an imbalance of these dual immune responses seems to be responsible for organ dysfunction. The pathogenesis of tissue injury is complex and cannot be attributed to a single agent. Tissue injury occurs during inflammation and is a progressive process which may eventually lead to organ dysfunction and failure. The systemic inflammatory response is the overproduction inflammatory reaction resulting from systemic mediator release that may lead to multiple organ dysfunctions (Figure 2). Excessive generation of proinflammatory signals can aggravate tissue damage because of the products derived from inflammatory cells. The increment of the levels of proinflammatory cytokines leads to activation of macrophages, neutrophils, NK cells, T cells, and B cells [67, 73, 111]. The inflammatory response is essential for structural and functional repair of injured tissue.

Among the clinical features observed in severely envenomed patients, mainly children, the gastrointestinal symptoms such as vomiting, excessive salivation, and abdominal pain have been frequently reported by several authors [112]. Based on these and others clinical observations, several studies have been carried out to improve our understanding of the effects of scorpion venom on the gastrointestinal system.

Experimental studies have shown that the injection of whole venom and purified toxins from the venom of scorpions can cause profuse salivation, increased gastric and pancreatic injuries as well as disorders of intestinal motility [113–117]. Most of these effects have been related to the acute autonomic disturbances triggered by the venom, which can provoke both the activation and delayed inactivation of neuronal sodium channels, where they modulate the release of neurotransmitters, that leads to a variety of adverse effects which include respiratory failure, lung edema, arrythmias, tachycardia followed by bradycardia, skeletal muscle stimulation, lacrimation, convulsions, and enlarged pupils, among others [112, 116–124]. However, the role of other members of IL-family in envenomation is increasingly appreciated, and in the present work are summarized all currently available information from human and experimental studies. With respect to the scorpion envenomation the immune response also is triggered by cascade that including the released of mediators such as nitric oxide and complement system.

*Nitric oxide (NO)* is a free radical generated by the conversion of the aminoacid L-arginine to NO and L-citrulline by the enzyme NO synthase and is the key endothelium-derived releasing factor implicated in the regulation of vascular tone and vasomotor function. NO is also considered as a second messenger for a number of physiological in processes including neurotransmission, maintenance of vasodilator tone, and arterial pressure and it has been suggested that cytokine-mediated circulatory shock is caused by activation of the inducible isoform of NOS [125]. Oneb of the isoforms, the endothelial NOS, plays an important role in determining and maintaining aspects of normal renal function, for instance proximal tubule sodium reabsorption. In biological systems such as cardiovascular and nervous, the NO has important functions by its implication as vasodilator and neurotransmitter. In some instances the NO decomposes to nitrite and nitrate, and cytokine-mediated increases in concentrations of nitrite/nitrate. NO is also involved in many pathophysiologic states, including insulin resistance. Several inducers of insulin resistance that including proinflammatory cytokines and oxidative stress are capable to cause modifications in the levels of nitrite and nitrate production have been associated with several conditions: severe envenomation, septic shock, and hypertension [83, 84]. NO plays a pivotal role in the pathophysiology and pathology in various systems including the envenomation caused by scorpion venom. The presence of NO in serum from patients envenomed with *L. quinquestriatus *and mice exposed to *T. serrulatus *venom, and supernatants of macrophage treated with *T. serrulatus *venom and/or and its toxins are shown in Table 5. The oxidant and anti-oxidant balance is an important determinant of immune cell function, not only for maintaining the integrity and functionality of membrane lipids, cellular proteins, and nucleic acids, but also for the control of signal transduction and gene expression in immune cells. The cells of the immune system are sensitive to changes in the balance of the oxidant/antioxidant because high levels of ROS are produced as part of their normal function.

One other effect is associated with complement-activating components in the venoms that indirectly contribute to tissue damage. Bertazzi et al., 2003 [126], described the effects of *T.serrulatus *venom and TsTX1 gamma on lytic activity of the complement system. In this study the authors showed that the complement system is involved in inflammatory process induced by the venom or toxin and consequently in the lung edema, hemolysis, leukocytosis, among other clinical manifestation of severe envenomation [127]. The effects of *Androctonus australis hector *venom on the lytic activity of the complement system were also studied and reported [87].

## 5. Organ Dysfunction and the Inflammatory Response

Scorpion envenoming essentially results in a syndrome of fuel-energy deficits and causes an inability to utilize the existing metabolic substrates by vital organs causing multi-organ system failure and death. The scorpion envenomation is associated with a pathophysiological manifestation. The effect of stings of scorpion venoms in humans and experimental animals exposed to venom, such as rabbits, rats, and mice were studied in serum biochemical parameters and are presented in Table 6. 

Paneque Peres et al. 2009 [135] shown that the Brazilian scorpion *T. serrulatus* resulted in increased lung, kidney, liver, and heart inflammation, characterized by an increased density of mononuclear cells after injection in rats. These authors concluded that this venom leads to acute lung injury, characterized by altered lung mechanisms and increased pulmonary inflammation. The primary pathological process is pulmonary capillary endothelial dysfunction resulting in interstitial and alveolar oedema of protein and phagocytic immune cell rich exudative fluid. Pulmonary oedema may develop rapidly after a sting. The symptoms associated with pulmonary oedema are variable but may be rapid. 

Depending on the severity and duration of the renal dysfunction this accumulation is accompanied by disturbances such as metabolic acidosis and hyperkalemia, changes in blood fluid balance, and effects on may other organ systems. The scorpion venom caused a great increase in renal oedema which is related to the decreased glomerular filtration rate and urinary flow. The *T. serrulatus* venom also affects haemodynamics probably by a direct vasoconstrictor action leading to increased renal flow in mice [136, 137]. Acute renal failure is a rapid loss of renal function due to damage to the kidneys, resulting in retention of nitrogenous such as creatinine and non-nitrogenous waste products that are normally excreted by the kidney. In various works the authors described that scorpion venom caused a great increase in renal oedema, which is related to the decreased glomerular filtration rate and urinary flow. During the acute renal failure has been reported to occur after scorpion stings [127, 138, 139]. In normal state the kidney maintains renal blood flow and glomerular filtration through autoregulation dependent on the tone of the afferent and efferent arterioles. The cytokine-induced systemic vasodilation and relative hypovolaemia in sepsis are responsible for renal hypoperfusion. The renal vasculature has been shown to participate variably to mediators of systemic vasodilation and renal blood. The kidney produces intrinsic vasoconstrictors in response to cytokines. In particular, the arachidonic acid metabolites of thromboxane and leukotrienes both reduce renal blood flow and antagonists of these substances have been shown to have renal protective effects. The kidney is susceptible to leukocyte mediated tissue injury with neutrophil aggregation in response to production of proteases and ROS. 

Fatality after scorpion envenomation may be the result of cardiovascular failure complicated by pulmonary oedema as well as by respiratory arrest. Both of heart and the blood vessels are sensitive to the effects of proinflammatory cytokines as well as vasoactive substances present in excessive amounts in envenomation. The response to fail is caused by an increase in cardiac output. A direct effect of scorpion venom on myocardium has been shown in several studies [128, 130, 131, 133, 140–142]. Aspartate aminotransferase (AST) is distributed to all parts of the body but mostly concentrates in the liver and the heart. The increment in its levels may be attributed to myocardial infarction or hepatic failure. The activity of alanine aminotransferase (ALT) is similar to AST that is considered a liver-specific enzyme. It increases more and remains longer than AST during hepatic failure or inflammation. Therefore, the increase in AST and ALT levels may be due to a direct action of the venom on the liver and the heart. Envenoming by different scorpions has showed an increase of circulating enzymes levels those are succinate dehydrogenase, creatine phosphokinase, lactate dehydrogenase (LDH), glucose-6-phosphate dehydrogenase (G6PD), ALT, and AST (Table 6).

Following venom injection the highest toxin concentration has been found in the kidneys, liver, heart, and lungs [128–134, 140–148]. The ALT and AST activities have reported in the serum change during scorpion envenomation [128–134, 140–149]. Scorpion venom also acts on the sodium-potassium pump, producing from local symptoms to systemic problems which lead to changes in the victim's life. Various studies described that the mechanisms of action toxins on cell membranes that producing changes on the transmembrane action potential and permeability changes in calcium and potassium channels altering the release of neurotransmitters such as acetylcholine [116]. Scorpion venom increases the membrane permeability to sodium by opening the voltage sensitive sodium channels, which is accompanied with calcium entry, and blockade of calcium-activated potassium channels resulting in relative hyperkalemia who induces the release of catecholamines. One of main metabolic changes produced by scorpion stings is hyperkalemia. In human victims of the Mexican scorpion there are reported the hyperkalemia and hyponatremia [117]. These authors described that hyponatremia could be explain abdominal distention caused by hypokalemic intestinal paralysis and hypernatremia, the cause of irritability. One factor that contributes and aggravates the hyperkalemia condition is that the potassium influx causes the pronounced and prolonged hyperglycemia, enhanced glycolysis, and inhibited glycogenesis from decreased insulin secretion. 

Some researchers have emphasized that blood glucose levels increase after envenomation resulting in hyperglycemia in animal models. Also several studies have reported that this might be to a massive release of catecholamines, increased glucagon and cortisol levels, changes in thyroid hormone levels and changes in insulin secretion [84, 87, 149]. The elevation of circulatory catecholamines and angiotensin result in intense vasoconstriction and cardiac stimulation, increased myocardial oxygen requirement, and alterations in myocardial perfusion and metabolism, with hyperglycaemia and an increase in circulating free fatty acids [149]. 

Murthy and Hase in 1994 [150] described that the insulin has a primary metabolic role in preventing and reversing the cardiovascular, haemodynamic, and neurological manifestations and pulmonary oedema induced by scorpion envenoming. Insulin is a pleiotropic hormone which has diverse functions including stimulation of nutrient transport into cells regulation of gene expression modification of enzymatic activity and regulation of energy homeostasis actions. Rabbits and dogs following scorpion envenomation present a reduction of insulin, hyperglycemia, and enlace glycogenolisis in heart, liver, and skeletal muscle [149, 150]. Various studies described the electrolytes changes such as occurrence of hyponatremia accompanied by normal to increased levels of serum potassium and lowered serum calcium after injection of scorpion venom from different species.

The production and/or release of cytokines may also play a role in the development of hyperglycemia, in particular TNF-*α* has been demonstrated to induce insulin resistance in animals models. Recent studies have showed that the proinflammatory cytokines, such as IL-1*β*, TNF-*α*, and IFN-*γ*, are putative mediators of the progressive loss of pancreatic *β*-cells in type diabetes mellitus. These cytokines are released by macrophages and T cells in infiltrated islets of Langerhans and cause impaired function and ultimately cell death by apoptosis or necrosis [151]. With respect to anti-inflammatory cytokines in particular IL-4, IL-10, and IL-13, is related to the protection of pancreatic *β*-cells [88, 152–157]. Cytokines acting alone or in combination induce various transcription factors and signal transduction pathways within *β*-cells. One of the most important signaling events is the activation of transcription factor, nuclear factor kappa B [157]. These factors play the role of a master switch in *β*-cells, activating transcription of a number of genes involved in cytokine-mediated toxicity. Of great importance for cytokine toxicity in *β*-cells are in particular the generation of nitric oxide via induction of the inducible nitric oxide synthase (iNOS) and production of reactive oxygen species. Cytokine-induced nitrosative and oxidative stresses trigger eventually *β*-cells death [153, 156, 157]. 

Although the action of anti-inflammatory cytokines has been studied in different cell types during recent years, little is known about the effects of these cytokines in pancreatic *β*-cells [158–161]. It is known from the studies in different cell models that IL-4 is able to counteract many of the IL-1*β* effects, and reduced NO production has been considered an important element for this beneficial effect [159, 162, 163]. The biological effects of IL-13 may be achieved through binding to the IL-4 receptor *α* and therefore it is generally assumed that these two cytokines overlap in the biological effects. The effects of IL-10 on the nitric oxide pathway are unclear and the opposite findings have been reported [162]. Some studies on anti-inflammatory cytokine action in insulin-producing cells have been published recently, but opposite effects with respect to *β*-cell survival have been reported and the underlying molecular mechanisms of the action of anti-inflammatory cytokines still remain unknown [163].

## 6. Conclusion

The mediators affecting inflammatory processes may be released after scorpion envenomation including kinins, ecosanoids, platelet activating factor, permeability increasing factor, nitric oxide, and cytokines. This released of cytokines and other mediators may account for several of inflammatory manifestations observed such as acute respiratory of inflammatory manifestations observed such as acute respiratory distress syndrome, systemic inflammatory responses syndrome and multiple organ failure. The cytokines regulate and amplify the immune response, induce tissue injury and mediate complications of the inflammatory response. Th1 cytokines are mainly proinflammatory, while Th2 cytokines are mainly anti-inflammatory. Equilibrium between pro- and anti-inflammatory is essential to maintain the homeostasis in the system. Dysregulations of the pro- versus anti-inflammatory are involved in the pathogenesis of envenomation in humans and experimental animals. The balance between proinflammatory and anti-inflammatory cytokines in envenomation determines the degree and extent of inflammation which can lead to major clinical effects such as cardiac dysfunction, pulmonary edema and shock. In line with the findings, high levels of TNF-*α*, IL-1*β*, IL-6 and IL-8 have also been reported after scorpion envenomation.

## Figures and Tables

**Table 1:** Neurotoxic and Cytotoxic local effects.

Neurotoxic	Cytotoxic
Local evidence of a sting may be minimal or absent in approximately 50% of cases of neurotoxic scorpion stings.	A macula or papule appears initially at the sting site, occurring within the first hour of the sting. If the lesion progresses to a purple plague that will necrosis and ulcerate.
Pain sensation at the sting site, followed by itch, erythema, local tissue swelling, and ascending hyperesthesia, that persists for several weeks, and is the last symptom to resolve before the victim recovers.	The diameter of the lesion is dependent on the quantity of venom injected.
	The progression of lesion to a purple plaque that will necrosis and ulcerate.
The site is hypersensitive to touch and temperature.	Lymphangitis results from the transfer of the venom through the lymphatic vessels.

**Table 2:** Central nervous system signs.

Signs	Characteristics
Sympathetic	Hyperthermia, tachypnea, tachycardia, hypertension, arrythimia, hyperkinetic pulmonary, oedema, hyperglucaemia, diaphoresis, piloerection, hyperexcitability, and convulsions.
Parasympathetic	Bronchoconstriction, bradycardia, hypotension, salivation, lacrimation, urination, diarrhea, priapism, dysphagia, and gastric emesis.
Somatic	Inactivation of sodium channels, increased tendon reflexes.
Cranial	Ptosis, dysphagia, pharyngeal reflex loss or muscle spasm.
Peripheral nervous system	Paralysis and convulsions.

**Table 3:** Grade of envenomation.

Grade	Characteristics
I	*Mild envenoming*
	Patients presenting only local symptoms, local pain, and a burning sensation.
II	*Moderate envenoming*
	Patients with local and general symptoms.
III	*Severe envenoming*
	Patients presenting with local and general symptoms, together with cardiocirculatory shock, respiratory failure, acute pulmonary edema, hyperthermia, and neurologic symptoms such as priapism, convulsions, and coma.

**Table 4:** Pro- and Anti-inflammatory cytokines.

Proinflammatory	Principal cells source	Biological activities
IL-1*α* and IL-1*β*	Macrophages and APC	Costimulation of APC and T cells inflammation and fever, acute phase reaction hematopoiesis.
IL-2	Activated Th1 and NK cells	Proliferation of B cells and activated T cells, NK functions.
IL-6	Activated Th2 cells, APC, other somatic cells	Acute phase reaction, thrombopoiesis, B cell proliferation, synergistic with IL-1 and TNF on T cells.
IL-8	Macrophages, other somatic cells	Chemoattractant for neutrophils and T cells.
TNF-*α*	Monocytes and macrophages	Wide ranging biological effects on lipid metabolism, coagulation and endothelial function. Induces inflammation and fever acute phase reaction, weight loss and antitumoral effects.
IFN-*γ*	Activated Th1 and NK cells	Induces of class I MHC on all somatic cells, induces class II MHC, an APC and somatic cells, activates macrophages, neutrophils, NK cells, promotes cell-mediated immunity antiviral effects.

Anti-inflammatory		

IL-4	Th2 and mast cells	B cell profileration, eosinophil and mast cell growth and function, IgE and class II MHC expression on B cells, inhibition of monokine production.
IL-10	Macrophages and lymphocytes	Exerts its anti-inflammatory activity by inhibiting TNF-induced NFkB activation.

**Table 5:** Mediators involved in envenomation.

Scorpion	Cytokines produced	References
Androctonus australis hector Experimental animal (rats)	IL-1*β*, IL-4, IL-6, IL-10, and TNF-*α*.	[87]

Buthus martensi Karch	NO and paw edema.	[88]

Centruroides noxius Experimental animal (mice)	IL-1*β*, IL-1*α*, IFN-*γ* IL-6, IL-10, and TNF-*α*.	[76]

Leiurus quinquestriatus Human and Experimental animal (rabbits)	IL-6, IL-8, NO, and TNF-*α*.	[86, 89, 90]

Tityus serrulatus Human and Experimental animal (rabbits)	IL-1*β*, IL-6, IL-8, IL-10, NO, TNF-*α*, IL-1*α*, IL-1*β*,IFN-*γ*, and GM-CSF.	[81–85, 91, 92]

**Table 6:** Metabolism.

Scorpion	Levels	References
Androctonus australis hector	LDH, CK.	[87]
Androctonus crassicauda	Glucose, cholesterol, ALT, AST, uric acid, bilirrubin, urea.	[128]
Hemiscorpious lepturus	ALT and AST.	[129]
Leiurus quinquestriatus	ALT and AST.	[130]
Odonthobuthus doriae	ALT and AST.	[131]
Palamneus gravimanus	ALT, AST, LDH, CK, uric acid, cholesterol, calcium, potassium.	[132]
Tityus serrulatus	Glucose, ALT, AST, CK, CXC, chemokine.	[83–85, 133, 134]
